# Supplementary material for: A Global Screen for Assembly State Changes of the Mitotic Proteome by SEC-SWATH-MS
Source: Cell Syst. 2020 Feb 26;10(2):133–155.e6. doi: 10.1016/j.cels.2020.01.001 (PMC7042714; doi:10.1016/j.cels.2020.01.001)

Q9NZJ9 | NUDT4_HUMAN | NUDT4 DIPP2 KIAA0487 HDCMB47P
Monomer MW [kDa]: 20.306 Monomer expected elution fraction: 54

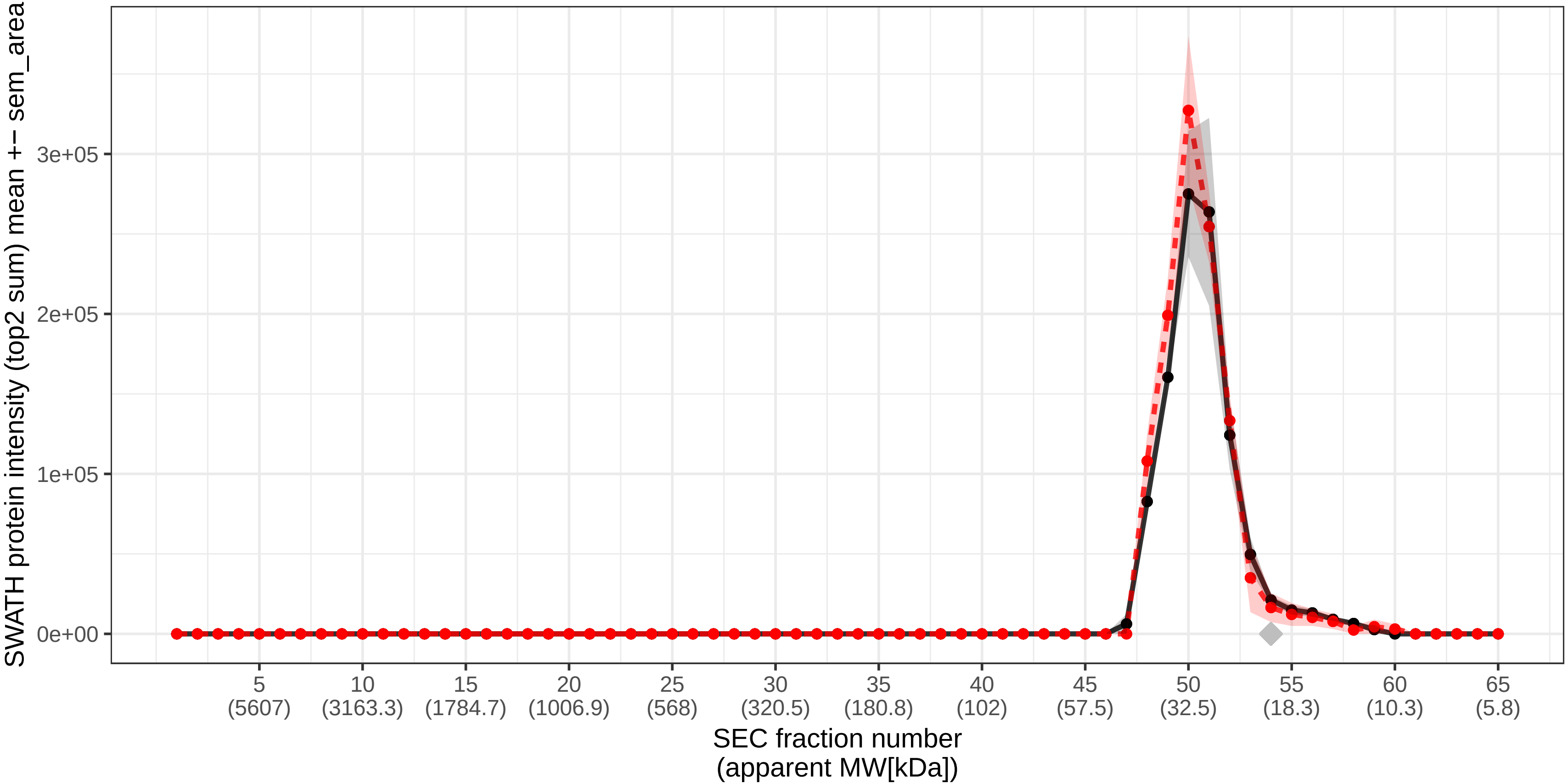

Supplement: Data S1. SEC-SWATH-MS Protein Chromatograms, Related to Figure 1 [file mmc6.zip › SECchrom_Q9NZJ9_NUDT4_HUMAN_NUDT4_DIPP2_KIAA0487_HDCMB47P.pdf]
